# A Non-Canonical Role for the Glycosyltransferase Enzyme UGT2B17 as a Novel Constituent of the B Cell Receptor Signalosome

**DOI:** 10.3390/cells12091295

**Published:** 2023-05-02

**Authors:** Antoine Wagner, Michèle Rouleau, Lyne Villeneuve, Trang Le, Cheryl Peltier, Éric P. Allain, Caroline Beaudoin, Sophie Tremblay, Fréderic Courtier, Flora Nguyen Van Long, Isabelle Laverdière, Éric Lévesque, Versha Banerji, Katrina Vanura, Chantal Guillemette

**Affiliations:** 1 Centre Hospitalier Universitaire de Québec Research Center—Université Laval (CRCHUQc-UL), Faculty of Pharmacy, and Centre de Recherche sur le Cancer de l’Université Laval (CRC-UL), Université Laval, Québec, QC G1V 4G2, Canada; 2 Department of Medicine I, Division of Haematology and Haemostaseology, Medical University of Vienna, 1090 Vienna, Austria; 3 Department of Internal Medicine & Biochemistry and Medical Genetics, Rady Faculty of Health Sciences, University of Manitoba, Winnipeg, MB R3E 3P4, Canada; 4 CancerCare Manitoba Research Institute, Cancer Care Manitoba, Winnipeg, MB R3E 0V9, Canada; 5 Molecular Genetics Laboratory, Dr. Georges-L-Dumont University Hospital Center, Moncton, NB E1C 2Z3, Canada; 6 CRCHUQc-UL, Faculty of Medicine, and CRC-UL, Université Laval, Québec, QC G1V 4G2, Canada

**Keywords:** glycosyltransferase, UGT2B17, CLL, B cell receptor, BTK, ZAP70, ibrutinib, cerdulatinib

## Abstract

In chronic lymphocytic leukemia (CLL), an elevated glycosyltransferase UGT2B17 expression (UGT2B17^HI^) identifies a subgroup of patients with shorter survival and poor drug response. We uncovered a mechanism, possibly independent of its enzymatic function, characterized by an enhanced expression and signaling of the proximal effectors of the pro-survival B cell receptor (BCR) pathway and elevated Bruton tyrosine kinase (BTK) phosphorylation in B-CLL cells from UGT2B17^HI^ patients. A prominent feature of B-CLL cells is the strong correlation of UGT2B17 expression with the adverse marker ZAP70 encoding a tyrosine kinase that promotes B-CLL cell survival. Their combined high expression levels in the treatment of naïve patients further defined a prognostic group with the highest risk of poor survival. In leukemic cells, UGT2B17 knockout and repression of ZAP70 reduced proliferation, suggesting that the function of UGT2B17 might involve ZAP70. Mechanistically, UGT2B17 interacted with several kinases of the BCR pathway, including ZAP70, SYK, and BTK, revealing a potential therapeutic vulnerability. The dual SYK and JAK/STAT6 inhibitor cerdulatinib most effectively compromised the proliferative advantage conferred by UGT2B17 compared to the selective BTK inhibitor ibrutinib. Findings point to an oncogenic role for UGT2B17 as a novel constituent of BCR signalosome also connected with microenvironmental signaling.

## 1. Introduction

Chronic lymphocytic leukemia (CLL) is the most prevalent type of leukemia in adults in Western countries [1]. It arises from mature malignant B-cells that progressively accumulate in the lymph nodes, bone marrow, spleen, and circulation. Enhanced proliferation rates, clonal growth of malignant B-cells, and defective cell death each contribute to the evolution of the disease. The clinical course of CLL is highly variable and comprises indolent and more aggressive subtypes identified using various biomarkers. These high-risk features include the mutational status of the immunoglobulin heavy chain variable (IGHV) gene, chromosome aberrations (del13q, trisomy 12, del11q, and del17p13), and immunophenotypic markers, such as CD38 and ZAP70, as well as many others that require further investigation [2,3]. These molecular markers help prognostication. The IGHV and chromosomal aberrations are also predictive and aid the selection of treatment type and duration to achieve the best outcomes for patients [4,5].

We and others have shown that an elevated glycosyltransferase *UGT2B17* expression identifies a subgroup of CLL patients with shorter treatment-free survival and overall survival, independent of other risk factors in multivariate analyses [6,7,8,9]. *UGT2B17* expression also represents a prognostic marker for this disease in CLL patients with mutated and unmutated IGHV [6,7,8]. UGT2B17’s high expression in high-risk patients further decreases response to fludarabine-containing treatments and is associated with resistance to targeted drugs, such as ibrutinib [8,10].

UGT2B17 is part of the 22 UGT metabolic enzymes best known for their key roles in regulating the bioactivity of endogenous molecules and in the inactivation of a wide variety of drugs, including anticancer agents from several classes [11]. UGT2B17 is the sole UGT enzyme significantly expressed in CLL cells and overexpressed in malignant B lymphocytes compared to healthy donors [8]. The molecular mechanism by which UGT2B17 influences CLL cell behavior and progression remains poorly understood. 

Overexpression of UGT2B17 in leukemic cell models promoted cell proliferation, supporting that UGT2B17 triggers pro-proliferative conditions [6]. The oncogenic functions of UGT2B17 are in part mediated by modulating the activity of signaling lipids and steroid hormones, with an influence on CLL progression [6,11,12]. UGT2B17 inactivates the signaling lipid prostaglandin E_2_ by conjugation to glucuronic acid and downregulates the prostaglandin signaling pathway, which displays tumor suppressor functions in B-cells [6]. UGT2B17 also contributes to the inactivation of antileukemic drugs, such as fludarabine [10], which was part of the antileukemic treatment arsenal used to treat CLL patients [4,5]. However, our recent findings indicated that UGT2B17 expression also conferred resistance to antileukemic drugs not conjugated by UGT2B17 [10], suggesting that functions independent of the conjugation activity of UGT2B17 are also at play in the context of CLL. 

On the basis of these recent observations, we hypothesized that UGT2B17 might influence the course of CLL by influencing the B cell receptor (BCR) signalosome. The BCR plays a central role in CLL as a proliferation and survival pathway, largely contributing to CLL progression. The BCR receptor triggers a cascade of molecular events mediated by a “BCR signalosome” comprised of signaling kinases, including the Bruton tyrosine kinase (BTK), targeted with ibrutinib used in frontline therapy [4,5]. We now report that UGT2B17 largely affects the expression and activity of the BCR signalosome. Mechanistically, the influence of UGT2B17 is likely independent of its enzyme function and mediated by protein-protein interactions with BCR proximal effectors, including BTK. Our observations further reveal crosstalk between UGT2B17, the BCR signalosome, and the interleukin/JAK/STAT pathway promoting the proliferation of CLL cells.

## 2. Materials and Methods

### 2.1. CLL Patient Cell Samples

Cryopreserved peripheral blood mononuclear cells (PBMCs) from four male and four female CLL patients who were diagnosed between 1987 and 2011 at Vienna General Hospital were studied for pSYK-Y352 levels using a specific BD Phosflow antibody (#557817, dilution 1:7; Becton, Dickinson, and Company, Franklin Lakes, NJ, USA). Cell lysates prepared from 22 male and 17 female CLL patients from the University of Manitoba Tumor Bank were studied for STAT6 (Cell Signaling Technology (Danvers, MA, USA) CST #9362) and pSTAT6-Y641 (CST #9361) by immunoblotting as described below. All patients provided written informed consent. The study was carried out in accordance with the Declaration of Helsinki and approved by the local ethical research committees of the Medical University of Vienna (Ethic vote 2176/2017), the University of Manitoba (REB#H2019288), and the CHUQc—Université Laval (#2015-1205).

### 2.2. Gene Expression Analysis

Publicly available expression data were obtained from 291 CLL cases of the International Cancer Genome Consortium (ICGC) dataset (project code CLLE-ES) [13] and from the Gene Expression Omnibus (GEO) accession numbers GSE66117, which included 48 cases [14], GSE13159 generated from PBMC of 448 treatment-naïve CLL patients [15], GSE15490 generated with CD19+-sorted B-cells from 20 CLL patients treated with fludarabine-containing regimens [16], GSE21029 from PBMC, lymph node and bone marrow cells of 24 treatment-naïve CLL patients [17], and GSE22529 from PBMC of 41 treatment-naïve CLL patients [18]. Differential gene expression analysis according to *UGT2B17* expression was performed using the DeSeq2 tool for R v3.2.2 [19] and considered significant if Benjamini-Hochberg adjusted *p* values were below 0.05. Spearman correlations between the expression of *UGT2B17* and *ZAP70* were determined using GraphPad Prism v9.0 (Dotmatics, San Diego, CA, USA). 

RNA sequencing data from MEC1 and JVM2 models have been described previously [6]. Pathway enrichment analysis was conducted with G: profiler (https://biit.cs.ut.ee/gprofiler/gost accessed on 31 March 2022) using Reactome, KEGG, and GO biological processes databases [20]. For redundant enriched pathways, only the most significant pathway names were retained in the final output. *ZAP70* mRNA expression was assessed by reverse transcription and quantitative real-time PCR (RT-qPCR) as described [6] and dichotomized at the median. Sequences of primers for ZAP70 amplification were Fw: 5′-GTT GAC TCA TCC TCA GAG ACG AAT C-3′ and Rev: 5′-AGG TTA TCG CGC TTC AGG AA-3′. The expression level of *UGT2B17* was determined by RT-qPCR as described, with UGT2B17^HI^ displaying ≥4.11 times the expression in healthy individuals [8]. RT-qPCR quantification was carried out using the StepOnePlus Real-time PCR System with PowerSYBR Green Master mix (Applied Biosystem, ThermoFisher, Waltham, MA, USA). Relative quantity was calculated using the ΔΔCt method. 

To assess the ZAP70 methylation status of 223 nucleotides downstream of the transcription start (*CpG*+*223*) [21], DNA was extracted from 5 × 10^6^ MEC1-CTRL and MEC1-UGT2B17^OE^ cells with the DNA QIAamp DNA Blood mini kit (Qiagen, Toronto, ON, Canada), and 1 µg was treated with the EpiTect bisulfite kit (Qiagen). PCR amplification of the ZAP70 CpG+223 region was achieved using AmpliTaq Gold 360 (ThermoFisher Scientific, St-Laurent, QC, Canada) using 100 ng DNA and the following primers: Fw 5′-TTT GTG ATT TTG ATG TAA A-3′ and Rv 5′-ACT CCC AAT TAA TAT TCT-3′, and the following PCR conditions: 95 °C for 10 min, 40 cycles of [95 °C for 30 s, 44 °C for 30 s, 72 °C for 30 s; 72 °C for 7 min]. A nested PCR was then performed using 1 µL of a 1:100 dilution of the first PCR reaction using the following primers: Fw 5′-AGG GTT ATT TTA GGG GGT TTT GGG-3′ and Rv 5′-CCC TAC AAC CCA AAC CCC C-3′, and the PCR conditions as above, except for the annealing temperature, which was 56 °C. PCR products were cloned using the QIAgen cloning kit, and at least 15 clones per cell line were Sanger sequenced to evaluate the methylation percentage and based on two biological replicates.

### 2.3. Cell Models and Cell-Based Assays

All cell models were obtained from DSMZ (Braunschweig, Germany). MEC1-CTRL, MEC1-UGT2B17^OE^, JVM2-CTRL, and JVM2-UGT2B17^OE^ were described previously, and overexpression of the UGT2B17 protein was confirmed by western blotting and functional assays, as in this previous report [6]. MEC-UGT2B17^KO^ and HG3-2B17^KO^ were generated by the CRISPR/Cas9 gene mutagenesis approach as described [22]. Functional UGT2B17 glucuronidation assays were conducted using dihydrotestosterone (DHT) as a substrate [10]. The knockdown (KD) of ZAP70 expression was achieved in MEC1-UGT2B17^OE^ and HG3 cells by transduction of three validated lentiviral pLKO.1-shRNA targeting human ZAP70 (Sigma-Aldrich, Oakville, ON, Canada). Lentiviral production, transduction, and selection with puromycin (MEC1-UGT2B17^OE^: 4 µg/mL; HG3: 0.5 µg/mL) was as described [22]. Cells transduced with TRCN0000000436 targeting the 3′-untranslated sequence 5′-CAGGCGTAGATCACCAGAATA of human *ZAP70* were used in subsequent analyses as they displayed the highest ZAP70 KD efficiency (>75%) confirmed by RT-qPCR and immunoblotting. Cell lines were grown in RPMI supplemented with 10% fetal bovine serum, 1% penicillin/streptomycin, 1% sodium pyruvate, and 1% L-glutamine. The growth medium of MEC1-UGT2B17^OE^ cells and MEC1-CTRL cells also contained 10 μg/mL blasticidin and 4 μg/mL puromycin, respectively. For JVM2-CTRL and JVM2-UGT2B17^OE^ cells, the growth medium was supplemented with 5 μg/mL blasticidin. For pharmacological treatments, 1 µM ibrutinib, 0.5 µM cerdulatinib (SelleckChem, Houston, TX, USA), or vehicle (0.2% DMSO) were added to the culture media for 24h. Cells were regularly tested for mycoplasma, with the most recent test carried out on 25 February 2023.

For proliferation assays, cells were plated at a cell density of 1 × 10^4^ cells/100 μL in 96-well plates. At each time point, 20 μL of CellTiter aqueous one solution cell proliferation reagent (MTS Promega, Madison, WI, USA) was added to each well. Absorbance at 490 nm was measured after a 4 h incubation at 37 °C, normalized with the values on the day of plating. Three independent replicates of cell viability assays were conducted and performed in triplicate. For ibrutinib or cerdulatinib treatments, cell viability was determined at day four in at least three independent assays. For calcium flux measurements based on two biological replicates, cells (1 × 10^6^/mL) were incubated for 30 min at 37 °C in a humidified incubator with 5% CO_2_ with 5 µM Fluo-4 (Invitrogen™ #F14201) and 0.02% pluronic acid (Sigma-Aldrich) in RPMI 1640. Cells were washed with PBS, then resuspended in 500 µL PBS for calcium measurement by cytometry on a BD FACSCelesta^TM^ instrument and the BD FACSDiva^TM^ software version 8.0.1.1 (Becton, Dickinson, and Company). Data analysis was performed using FlowJo V10.8.1 (Ashland, Or, USA).

### 2.4. Antibodies and Immunoblotting

Protein cell lysates were prepared in lysis buffer (50 mM Tris-HCl, pH 7.4, 150 mM NaCl, 0.3% deoxycholate, 1% IGEPAL, 1 mM EDTA, COMPLETE anti-protease (Sigma-Aldrich), and PhosSTOP anti-phosphatase (Sigma-Aldrich)). Protein concentration was measured by a bicinchoninic acid assay (Thermo Scientific). Proteins were detected by SDS-PAGE, immunoblotting, and chemiluminescent detection with Clarity or Clarity MAX reagents using a ChemiDoc Imaging system (Bio-Rad Laboratories, Mississauga, ON, Canada). Antibodies used were as follows: UGT2B17: pan UGT2B EL93; EL-UGT2B17mAb [23]; LYN: CST #4576; BTK: CST #8547; pBTK-Y223: CST #5082; ZAP70: CST #2709; SYK: CST #13198; pSYK-Y352/pZAP-Y319: CST #2701; BLNK: CST #36438; pBLNK-Y96: CST #3601; STAT6: CST #9362; and pSTAT6-Y641: CST #9361, dilution 1:1000. Detection of GAPDH (Sigma-Aldrich G8795, 1:20 000), tubulin: CST #2144, 1:1000, vinculin: Sigma-Aldrich #05-386, clone V284, 1:5000, or total protein stain (TCE): 2,2,2-Trichloro-ethanol (Sigma-Aldrich, 1:200) on the same blots were used to normalize quantitative protein assessments between samples.

### 2.5. Immunoprecipitation

MEC1-UGT2B17^OE^, JVM2-UGT2B17^OE^, and HG3 cells (20 × 10^6^ per immunoprecipitation) were lysed in IP Buffer (40 mM HEPES, pH 7.5, 120 mM NaCl, 0.3% CHAPS, 0.3% sodium deoxycholate, 1 mM EDTA, 1 mM DTT, and COMPLETE protease inhibitors as previously described [24]). UGT2B17 was immunoprecipitated overnight with the pan-UGT2B EL-93 (1:400). Rabbit IgG immunoglobulins (1:400; Sigma-Aldrich #I5006) were used in control immunoprecipitations. Protein complexes were isolated using Dynabeads-protein G (Invitrogen) and washed in IP Buffer. Protein complexes were eluted by heating in Laemmli buffer and resolved by SDS-PAGE. Protein detection was by immunoblotting with antibodies described above.

### 2.6. Immunofluorescence

HG3 cells (2 × 10^6^/mL) were plated on poly-L-lysine coated coverslips in phosphate-buffered saline (PBS) and incubated for 1 h at 37 °C [25]. Cells were washed in PBS, fixed in 4% formaldehyde for 15 min, permeabilized with 0.25% Tween 20 in PBS for 4 min, and then washed in PBS. Cells were incubated overnight with ZAP70 antibody (1:400, CST #2709) and EL95 (1:750; [26]) or with BTK antibody (1:400, CST #8547) and EL-UGT2B17mAb (1:750) in a humid chamber at 4 °C, washed with PBS-Tween 20 (0.01%), and incubated for 1 h with goat anti-rabbit AlexaFluor 488 and goat anti-mouse AlexaFluor 555 (each 1:1000, ThermoFisher Scientific). Nuclei were stained with DRAQ5 (1:5000, Abcam, Toronto, ON, Canada), then cells were mounted with Fluoromount (Southern Biotech, Birmingham, AL, USA). Visualization and image acquisition were conducted on an LSM510 META NLO laser scanning confocal microscope (Zeiss, Toronto, ON, Canada) operated with the Zen2009 software version 5.5SP1 (Zeiss). Co-localization profiles were produced using the Fiji software (release 2.1.1.0).

### 2.7. Protein Stability

Cells (10 × 10^6^) were plated at a density of 4 × 10^5^ cells/mL in a T75 flask and grown for 48 h, after which the growth medium was replaced with a medium containing 20 μg/mL cycloheximide (Sigma-Aldrich) or DMSO (control) at selected time points, performed in biological duplicates. Cells were collected by centrifugation, washed in PBS, and lysed in Lysis Buffer. Protein half-life was assessed by immunoblotting as above.

## 3. Results

### 3.1. Interplay between UGT2B17 and the BCR Signalosome

**Figure 1:**
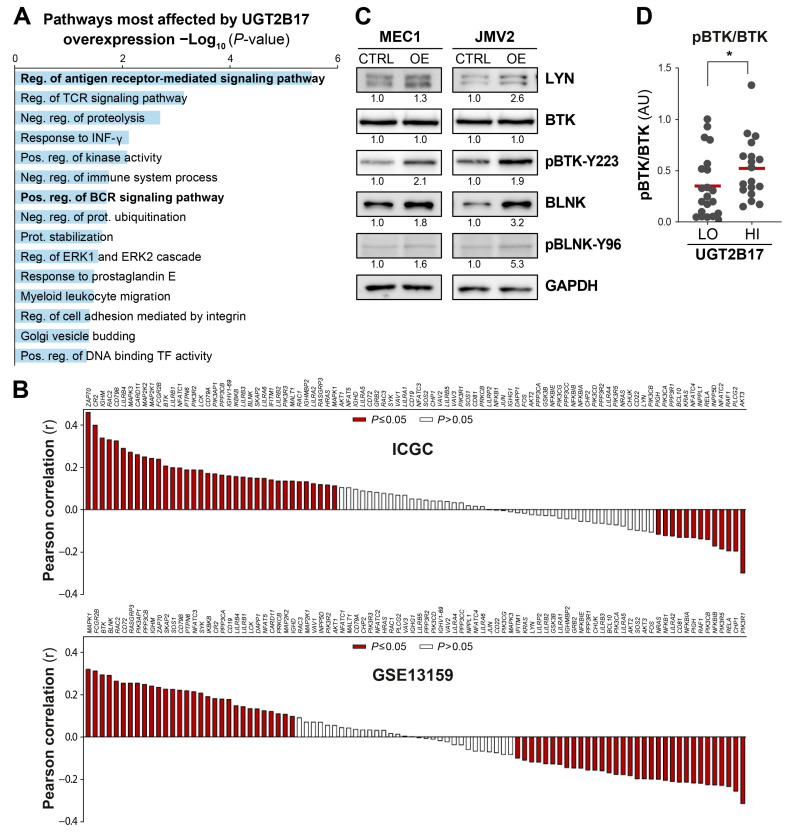
UGT2B17 expression level influences proximal BCR signaling effectors. (**A**) Pathway enrichment analysis with the 683 genes commonly and significantly changed in MEC1 and JVM2 leukemic models overexpressing UGT2B17. Pos: positive; Neg: negative; Reg: regulation; IFNγ: interferon gamma; BCR: B-cell receptor; TCR: T-cell receptor; and TF: transcription factor. (**B**) Pearson correlations of *UGT2B17* expression with genes related to BCR signaling pathway in two large CLL cohorts. For each cohort, genes were ranked from most positively correlated to most negatively correlated. (**C**) Expression of proximal BCR signaling effectors is enhanced in UGT2B17^OE^ leukemic cell models. (**D**) pBTK is higher in UGT2B17^HI^ CLL patients, revealed by WB. LO, HI: patients’ cells with low or high *UGT2B17* expression levels assessed by RT-qPCR as specified in Methods. *, *p* ≤ 0.05.

Transcriptome profiling of neoplastic B-cell models overexpressing UGT2B17 (UGT2B17^OE^) and CLL patients with high UGT2B17 expression (UGT2B17^HI^) demonstrated a considerable transcriptional shift in genes regulating proliferation and survival signaling pathways (Figure 1). Notably, a pathway enrichment analysis of the perturbed gene expression identified the “regulation of antigen-receptor mediated signaling pathway” as one of the most significantly enriched pathways in cell models (Figure 1A). The expression of several key pro-survival components of BCR signaling was correlated with the expression of *UGT2B17* in CLL patients, including *BTK, BLNK,* and *ZAP70* among the most positively linked to *UGT2B17* expression (Figure 1B). At the protein level, overexpression of UGT2B17 in cell models triggered an overexpression of LYN and BLNK and enhanced proximal signaling based on ~2-fold higher ratios of pBTK-Y223/BTK and pBLNK-Y96/BLNK (Figure 1C). A greater BCR-signaling capacity was further demonstrated in circulating B-CLL cells from UGT2B17^HI^ patients using pBTK-Y223 protein as a biomarker, showing a greater pBTK/BTK signal (by 1.5-fold; *p* = 0.037) compared to UGT2B17^LOW^ patients (Figure 1D). The enhanced phosphorylation of key BCR signaling proteins, such as BTK, implies that higher UGT2B17 expression levels improve the activation status of the BCR signalosome. This was also supported by measuring intracellular calcium mobilization [27], a surrogate marker of the BCR signalosome activity (Figure S1). This first set of data supports an interplay between UGT2B17 and the BCR signalosome at the transcriptional, translational, and posttranslational levels both in CLL patients’ cells and leukemic cell models and implies that UGT2B17 might prime the BCR pathway leading to the increased BCR signaling known to increase proliferation and survival of B-cells and more active disease.

### 3.2. Correlation of UGT2B17 Expression with the Adverse Marker ZAP70 and the Influence of Their Co-Expression on the Overall Survival of CLL Patients

**Figure 2:**
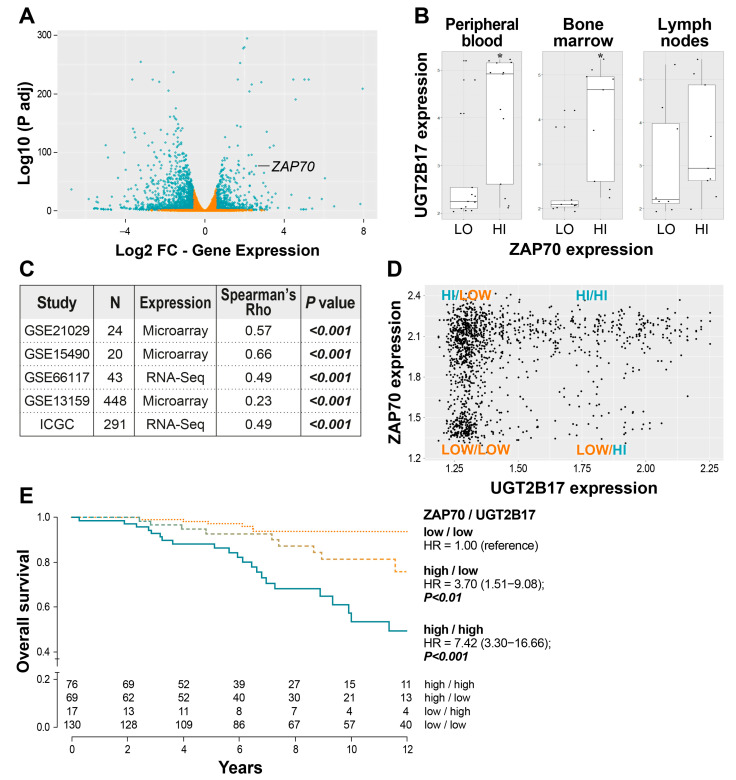
Association between the expression of poor prognosis genes *UGT2B17* and *ZAP70* in CLL patients. (**A**) RNA-seq-based differential gene expression identifies *ZAP70* as one of the most upregulated genes in leukemic UGT2B17 OE cell models relative to CTRL. Differentially expressed genes (fold change <−1.3 or > 1.3 (false discovery rate < 0.05) are represented by blue points. (**B**) UGT2B17 expression is higher in ZAP70^HI^ cells of CLL patients isolated from peripheral blood (*, *p* < 0.05), bone marrow, and lymph nodes. (**C**) Significant association between the expression of *UGT2B17* and *ZAP70* in five independent CLL cohorts [13,14,15,17,18]. (**D**) Distribution of CLL cases (GSE13159) according to *UGT2B17* and *ZAP70* expression levels delineates subgroups of CLL patients. (**E**) Survival outcome of CLL cases of the ICGC cohort based on *UGT2B17* and *ZAP70* expression levels. The number of cases was insufficient in the UGT2B17^HI^/ZAP70^LOW^ group to evaluate survival risk.

One of the BCR components persistently overexpressed in UGT2B17^HI^ patients and cell models compared to UGT2B17^LOW^ was ZAP70 (Figure 1B, Figure 2A, and Figure S2A), which is known to enhance BCR signaling [28,29,30]. To evaluate the relationship between the expression of *UGT2B17* and *ZAP70*, we initially studied the GSE21029 data set that provides gene expression from purified tumor cells obtained concurrently from blood, bone marrow, and lymph nodes of CLL patients. Data exposed that *UGT2B17* expression was increased along with higher *ZAP70* expression in matched paired samples of all three sites (Figure 2B). Additional evaluation of five independent CLL cohorts supported a significant correlation between the expression of both genes with significant Spearman coefficients (*p* < 0.001) (Figure 2C). A subsequent analysis uncovered different groups based on the expression of both markers (Figure 2D), associated with variable survival outcomes in treatment-naïve patients (Figure 2E). Different prognostic groups were defined with cases displaying a high expression of *ZAP70* and *UGT2B17* (UGT2B17^HI^/ZAP70^HI^) having the highest risk of poor survival with a hazard ratio (HR) value of 7.42 (95% confidence interval (CI) = 3.30−16.66); *p* < 0.001 compared to the reference group presenting the low expression of both genes (UGT2B17^LOW^/ZAP70^LOW^) (Figure 2E). Data supports that the combined expression of *UGT2B17* and *ZAP70* leads to poor outcomes. 

Previous studies demonstrated that methylation of a CpG island, 223 nucleotides downstream of the ZAP70 transcription start site (CpG+223), was critical in *ZAP70* transcriptional regulation and predicted the survival outcome in CLL patients [21,31]. We observed a significant induction of ZAP70 expression, both at the mRNA and protein levels, following overexpression of the UGT2B17 protein in the MEC1 cell line that displays a weak basal expression of ZAP70 undetected by western blot (Figure 2A and Figure S2A). Investigations of the methylation status of the CpG+223 site of the *ZAP70* gene revealed the absence of methylation in the MEC1-UGT2B17^OE^ cells consistent with high ZAP70 expression, whereas control cells were methylated at the CpG+223 site consistent with low ZAP70 expression (Figure S2B,C). This result suggests that UGT2B17 expression influences the epigenetic regulation of the *ZAP70* gene.

### 3.3. The UGT2B17-Dependent Enhanced Proliferation of Leukemic Cells Is Impaired by ZAP70 Depletion

**Figure 3:**
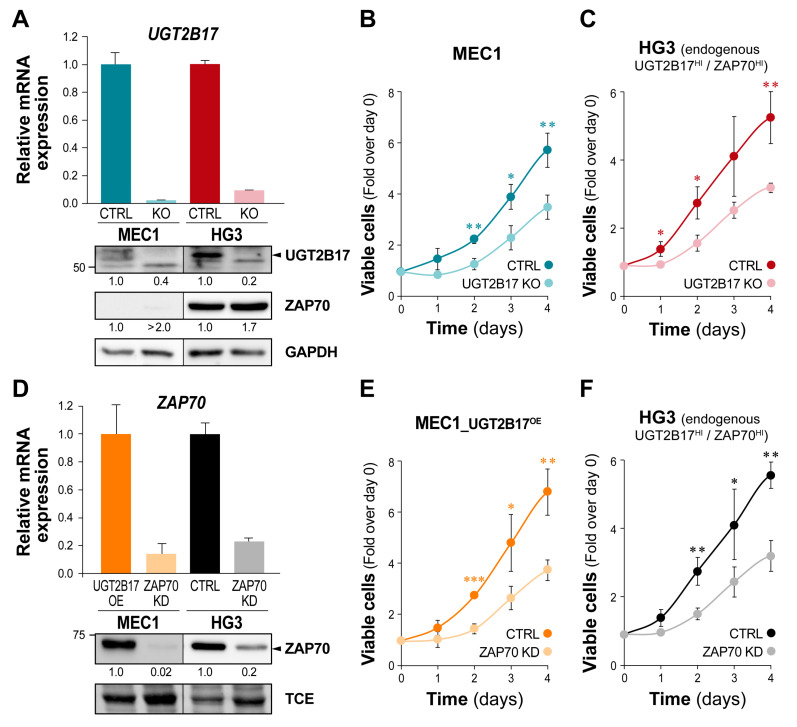
The UGT2B17-dependent enhanced proliferation of leukemic cells is impaired by ZAP70 depletion. (**A**) Relative UGT2B17 and ZAP70 expression in MEC1 and HG3 control (CTRL) and *UGT2B17* gene knockout (KO) cells. (**B**,**C**) UGT2B17 KO significantly reduces growth of (**B**) MEC1 and (**C**) HG3 cells. MTS absorbance was normalized to that at time of plating, based on biological replicates (n = 2). (**D**) Relative ZAP70 expression in MEC1-UGT2B17^OE^ and HG3 is genetically modified to repress ZAP70 (KD) with shRNAs. TCE: total protein loading control. (**E**,**F**) The repression of ZAP70 expression (KD) significantly reduces growth of (**E**) MEC1-UGT2B17^OE^ and (**F**) HG3 cells. Endogenous expression of UGT2B17 is naturally high in the CLL cell line HG3. *p*-values indicated by *, **, and *** correspond to <0.05, <0.01, and <0.001, respectively.

To explore the impact of UGT2B17 and ZAP70 expression levels on leukemic cell behavior, we conducted proliferation assays in leukemic B cell models. First, the *UGT2B17* gene was disrupted by CRISPR/Cas9 to create knockout (KO) cell models. The silencing of UGT2B17 expression was confirmed at the mRNA level (>90%) and protein level (>60%) (Figure 3A) and abolished UGT2B17 enzyme activity in both cell models (*p* < 0.001; Figure S3). The expression of ZAP70 in *UGT2B17* KO was similar to control cells (Figure 3A). Compared to the reference cells, silencing of the *UGT2B17* gene led to significant inhibition of cell proliferation by nearly 40% at day four (*p* ≤ 0.01) for both models (Figure 3B,C). Then, abrogation of ZAP70 was engineered by shRNA in MEC1 overexpressing ectopic UGT2B17 (MEC1-UGT2B17^OE^) and in the HG3 cell line endogenously expressing high levels of UGT2B17. Both models displayed significant repression of ZAP70 by >75% at the mRNA and protein levels, validating ZAP70 knockdown (KD) (Figure 3D). ZAP70^KD^ resulted in a markedly decreased cell proliferation of MEC1-UGT2B17^OE^ cells by nearly 50% after two to four days (*p* < 0.01; Figure 3E). Likewise, ZAP70^KD^ in HG3 led to a reduction of proliferation by 28% after 24 h and over 40% after 48 h (*p* < 0.01), compared to control cells (Figure 3F). Data indicate that both UGT2B17 and ZAP70 influence cell proliferation and support an effect of UGT2B17 on the BCR signaling that might involve ZAP70.

### 3.4. UGT2B17 Interacts with Proximal BCR Effector Proteins in Leukemic Cells

**Figure 4:**
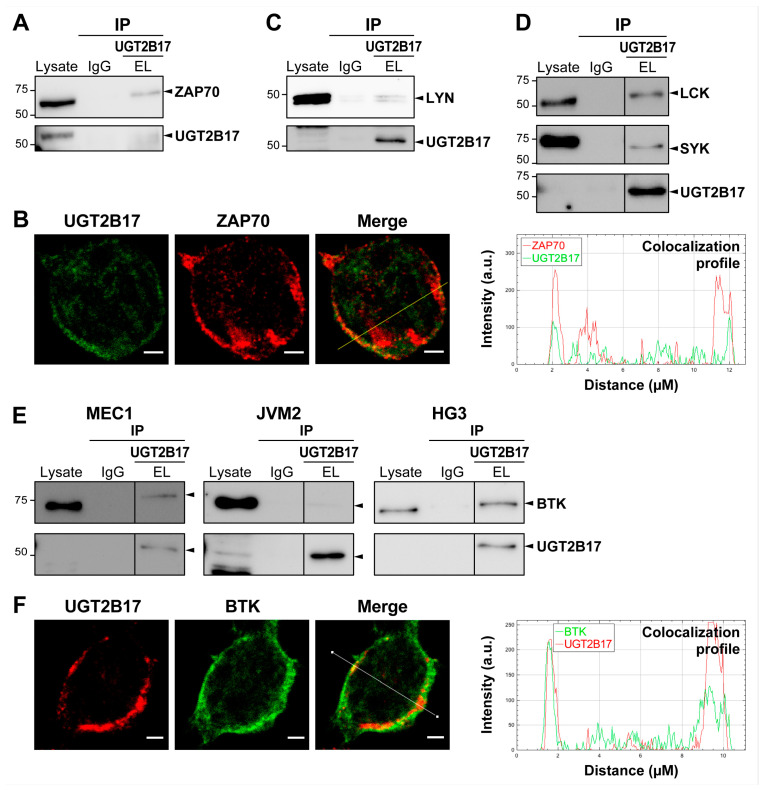
UGT2B17 interacts with proximal BCR effector proteins in CLL cell models. (**A**) Immunoprecipitation of endogenous UGT2B17 in HG3 specifically co-precipitates ZAP70. (**B**) Co-localization of UGT2B17 and ZAP70 in HG3 demonstrated by confocal immunofluorescence detection and further shown in the cross-sectional labeling intensity profile. (**C**,**D**) Immunoprecipitation of endogenous UGT2B17, specifically co-precipitates (**C**) LYN, (**D**) LCK, and SYK, shown in HG3 cells. (**E**) UGT2B17 interacts with BTK, as shown in MEC1-UGT2B17^OE^, JVM2-UGT2B17^OE^, and HG3. (**F**) Co-localization of UGT2B17 and BTK in HG3 cells demonstrated by confocal immunofluorescence detection and further shown in the cross-sectional labeling intensity profile. The detection of UGT2B17 by immunoblotting in cell lysates often provides faint signal that is enhanced by immunoprecipitation. IP: immunoprecipitation; IgG: isotype control immunoglobulins; and EL is an anti-UGT2B antibody.

We then reasoned that UGT2B17 might potentiate BCR signaling by interacting with BCR effector proteins, such as ZAP70. To test this hypothesis, we evaluated the potential of UGT2B17 to interact with ZAP70 and proximal BCR effectors based on immunoprecipitation of UGT2B17 in cell lines expressing high levels of UGT2B17. ZAP70 was identified as a novel protein partner of UGT2B17 shown in HG3 expressing high endogenous levels of UGT2B17 (Figure 4A). Then, we performed confocal microscopy to validate that leukemic cells co-express intracellular UGT2B17 and ZAP70. Merged images supported the colocalization of these proteins, with further additional evidence being provided by the colocalization profile revealing an enrichment of the merge signal at the plasma membrane where the BCR is expressed (Figure 4B). An interaction of UGT2B17 with additional components of the BCR pathway was observed, including with the proto-oncogene tyrosine kinase LYN also essential to CLL progression, the spleen tyrosine kinase (SYK) closely related to ZAP70, as well as with the lymphocyte-specific protein *tyrosine kinase* and activator of ZAP70, and LCK (Figure 4C,D). Lastly, the central component of BCR signaling, BTK, which regulates B cell proliferation and survival, was also specifically co-purified with UGT2B17, supporting interaction with BTK as well (Figure 4E). Colocalization of endogenous UGT2B17 and BTK was also shown in HG3 (Figure 4F). Additionally, data does not support that UGT2B17 expression levels affect the stability of this newly identified interactor nor of ZAP70 (Figure S4).

### 3.5. Efficient Targeting of the UGT2B17 Pro-Proliferative Effect by Dual Inhibition of SYK/JAK Kinases

**Figure 5:**
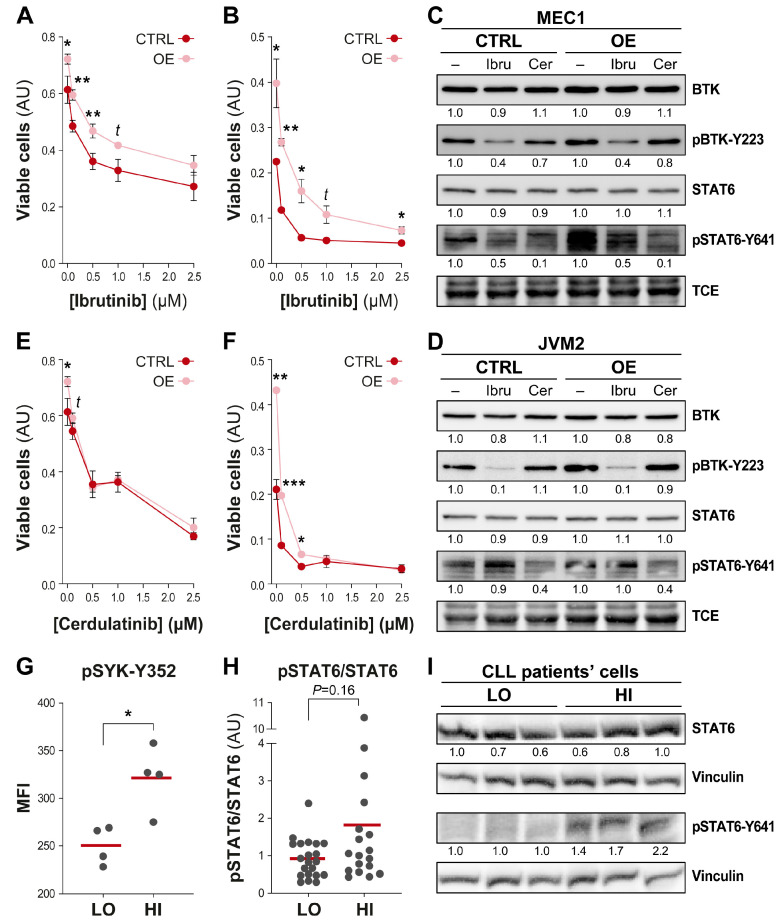
The proliferative advantage conferred by UGT2B17 is compromised by the dual JAK/STAT and SYK inhibitor cerdulatinib. (**A**,**B**). (**A**) MEC1-UGT2B17^OE^ and (**B**) JVM2-UGT2B17^OE^ cells retain their proliferative advantage over control (CTRL) cells when exposed to the BTK inhibitor ibrutinib. (**C**,**D**). Expression and phosphorylation (p) of BTK and STAT6 in (**C**) MEC1 and (**D**) JVM2 cells following a 24 h exposure to 1 µM ibrutinib and 0.5 µM cerdulatinib. TCE: total protein loading control. (**E**,**F**). The proliferative advantage of (**E**) MEC1-UGT2B17^OE^ and (**F**) JVM2-UGT2B17^OE^ cells is compromised by cerdulatinib. (**G**) UGT2B17^HI^ CLL patient cells have a significantly higher level of pSYK-Y352, assessed by phosphoFlow (n = 8). (**H**) UGT2B17^HI^ CLL patient cells tend to have a higher level of pSTAT6 levels (n = 39). (**I**) Expression of STAT6 and pSTAT6 assessed in B-cells from six representative CLL UGT2B17^LOW^ (n = 3) and UGT2B17^HI^ (n = 3) cases, based on *UGT2B17* expression levels assessed by RT-qPCR as specified in Methods. *, *p* ≤ 0.05; **, *p* ≤ 0.01; ***, *p* ≤ 0.001; *t*, trend.

Altogether, data demonstrate that UGT2B17 interacts with several kinases of the BCR signaling pathway in a constitutive manner and imply that the UGT2B17 protein could promote CLL progression independently of its transferase activity through functional association with BCR-related proteins. This also raises the possibility that targeting BTK might inhibit UGT2B17’s pro-proliferative effect. We selectively inhibited BTK using ibrutinib in UGT2B17^OE^ cell models (MEC1-UGT2B17^OE^ and JVM2-UGT2B17^OE^) and compared cell proliferation to control cells. A significant proliferative advantage was maintained for ibrutinib-treated UGT2B17^OE^ cells, suggesting that BTK inhibition was not sufficient to abrogate the pro-proliferative advantage conferred by UGT2B17 (Figure 5A,B). In these conditions, we confirmed that BTK phosphorylation was blocked (Figure 5C,D) [32].

This suggested that inhibition of SYK may be a potential therapeutic strategy to alleviate the UGT2B17 pro-proliferative effects that were not compromised by ibrutinib treatment. To inhibit SYK, we selected cerdulatinib, which antagonizes both BCR and interleukin signaling pathways by inhibiting SYK and the JAK1/3-STAT6 pathway [33,34]. As shown in Figure 5E,F, UGT2B17^OE^ cells were more sensitive to cerdulatinib, a finding supported by repression of JAK1/3-mediated phosphorylation of STAT6 (pSTAT6) (Figure 5C,D). Coherent with this concept, UGT2B17^HI^ B-CLL patient cells displayed greater activation of SYK (1.3-fold; *p* = 0.03) (Figure 5G), as well as pSTAT6, that did not reach significance, compared to UGT2B17^LOW^ CLL cases (Figure 5H,I). Data supports the idea that the dual inhibition of SYK/JAK kinases may be a therapeutic strategy to overcome the UGT2B17 pro-proliferative effect.

## 4. Discussion

The independent prognostic feature of high *UGT2B17* gene expression in B-CLL cells demonstrated in independent patient cohorts was unexpected for a detoxification enzyme [6,7,8] and led us to postulate an influence on the BCR signaling pathway fundamental in promoting CLL progression. Here, we provide compelling evidence that the high expression of the UGT2B17 protein enhances the BCR-signaling capacity in B-CLL cells and promotes proliferation through functional interplay with several BCR effectors, including BTK, and with other activating signaling pathways, such as those triggered by cytokines. UGT2B17^HI^ patients’ cells and cell models expressing high levels of UGT2B17 displayed greater expression and activity of pro-oncogenic signaling kinases, such as ZAP70, pBTK, and pBLNK of the BCR pathway. The enhanced proliferation of UGT2B17 overexpressing cells was more effectively abrogated by the dual SYK/JAK inhibitor cerdulatinib compared to ibrutinib targeting BTK, supporting that the oncogenic functions of UGT2B17 are also mediated by the JAK/STAT signaling pathway. Overall, data suggest a non-canonical role for UGT2B17 as a novel constituent of BCR signalosome and its connection to the IL4 cytokine signaling pathway. 

Gene expression profiling of CLL patient cohorts revealed that *UGT2B17* overexpression is associated with the enhanced expression of several key pro-survival components of BCR signaling. This is in keeping with the transcriptional rewiring triggered by UGT2B17 overexpression in leukemic cells, which significantly affected immune cell signaling genes [6,34], and may be in part mediated by epigenetic changes, as suggested by our observations for ZAP70 methylation. Our findings suggest that a greater BCR-signaling capacity may prevail in UGT2B17^HI^ patient cells, a notion supported by the elevated levels of phosphorylated BTK protein in UGT2B17^HI^ CLL patient cells compared to UGT2B17^LOW^. In leukemic cell models, expression and kinase activity of several proximal BCR effectors were enhanced by overexpression of UGT2B17, including the T-cell receptor effectors LCK and ZAP70, which are documented BCR signaling effectors in CLL cells [28,35]. 

ZAP70 is a poor prognostic marker known to enhance BCR signaling and was one of the most perturbed genes associated with *UGT2B17* overexpression. *ZAP70* expression was tightly correlated with *UGT2B17* expression in several independent CLL cohorts. Although high expression of each marker predicted poor survival in treatment-naïve cases, the combined expression of *UGT2B17* and *ZAP70* defined a more aggressive disease with the highest risk of shorter survival amongst all expression subgroups, suggesting a cumulative prognostic impact on survival outcome. Our result also suggests that UGT2B17 expression may influence the epigenetic regulation of the *ZAP70* gene by a mechanism that remains to be elucidated. In leukemic B-cells, a knockout of UGT2B17 expression significantly reduced proliferation, in keeping with our previous data, indicating that high UGT2B17 expression promoted B-cell proliferation [6]. Repression of ZAP70 in UGT2B17^OE^ cells also reduced the proliferative capacity of B-cells, further demonstrating that the pro-proliferative effect of UGT2B17 involved ZAP70 expression.

Mechanistically, we provide evidence that the UGT2B17 enzyme forms a complex with ZAP70, as well as with multiple other BCR effector kinases, namely LCK, LYN, SYK, and BTK. In a recent investigation in CLL, patient cells showed constitutive protein interactions between ZAP70, BTK, and SYK [36], and our data imply that UGT2B17 is a part of this complex, suggesting a non-enzymatic role for UGT2B17. It remains to be defined whether UGT2B17 is a target of these kinases and/or whether UGT2B17 acts as a scaffolding protein facilitating the function of the BCR signalosome. In support of this notion, a recent study highlighted a mechanism by which the interaction of UGT2B28 with the adaptor huntingtin interacting protein 1 triggered epidermal growth factor *receptor* signaling to promote prostate cancer cell proliferation and invasion [24]. Despite the fact that little is known about the UGT domains involved in protein interactions, multiple reports argue in favor of UGTs being a part of functional protein complexes unrelated to their known metabolizing functions [37,38,39,40]. This adds to the previously documented inactivation of prostaglandin E_2_ by UGT2B17-mediated glucuronidation that abrogated the tumor suppressor functions of PGE_2_ in B-cells and exposed an enzymatic mechanism by which UGT2B17 might influence CLL progression [6].

Collectively, our observations suggest that elevated UGT2B17 expression potentiates oncogenic BCR signaling leading to the enhanced proliferation of B-CLL cells. In a previous study, we showed that high UGT2B17 in B-cell models led to reduced sensitivity to ibrutinib, which is not explained by UGT2B17-meditated inactivation of ibrutinib [10]. SYK plays a central role in the transmission of activating signals downstream of the BCR, as well as chemokine and integrin receptors within B cells. The inhibition of SYK was shown to overcome resistance to ibrutinib in vitro, indicating that inhibition of SYK may be a therapeutic strategy to overcome the UGT2B17 pro-proliferative effect [41]. Although inhibition of BTK did impair proliferation in leukemic cell models, a greater sensitivity to dual inhibition of SYK and JAK1/3 by cerdulatinib that antagonizes BCR and microenvironmental signaling suggests an interplay between UGT2B17 and the interleukin/JAK/STAT6 pathway. This notion was also supported by the enhanced pSYK and pSTAT6 levels in primary UGT2B17^HI^ CLL patient cells. The JAK/STAT6 pathway may, thus, further contribute to UGT2B17 oncogenic functions, and our results imply that it could constitute a therapeutic vulnerability in UGT2B17^HI^ CLL cases.

## 5. Conclusions

This work expands our current knowledge regarding the function of UGT2B17 in CLL by placing this protein as a component of the BCR signalosome and at the crossroad of the BCR signalosome and the interleukin/JAK/STAT6 pro-survival pathways.

## Data Availability

The original contributions presented in the study are included in the article/supplementary material. Further inquiries can be directed to the corresponding author.

## Supplementary Materials

The following supporting information can be downloaded at: https://www.mdpi.com/article/10.3390/cells12091295/s1. Figure S1: Intracellular calcium mobilization is higher in UGT2B17^OE^ leukemic cells. Figure S2: High UGT2B17 expression is associated with higher ZAP70 expression and reduced methylation of the *ZAP70* 5′ regulatory region. Figure S3: Validation of MEC1-UGT2B17^KO^ and HG3-UGT2B17^KO^ models by a specific enzymatic assay. Figure S4: The stability of BCR effectors is not compromised by the expression of UGT2B17.
