# Supplementary material for: A Non-Canonical Role for the Glycosyltransferase Enzyme UGT2B17 as a Novel Constituent of the B Cell Receptor Signalosome
Source: Cells. 2023 May 2;12(9):1295. doi: 10.3390/cells12091295 (PMC10177405; doi:10.3390/cells12091295)
Supplement: Supplementary file 1 [file cells-12-01295-s001.zip › cells-2341733-supplementary.pdf]

Supplementary Figures

Figure S1: Intracellular calcium mobilization is higher in UGT2B17OE leukemic cells.
Figure S2: High UGT2B17 expression is associated with higher ZAP70 expression and reduced methylation of the ZAP70 5′ regulatory region.
Figure S3: Validation of MEC1-UGT2B17KO and HG3-UGT2B17KO models by a specific enzymatic assay.
Figure S4: The stability of BCR effectors is not compromised by the expression of UGT2B17.

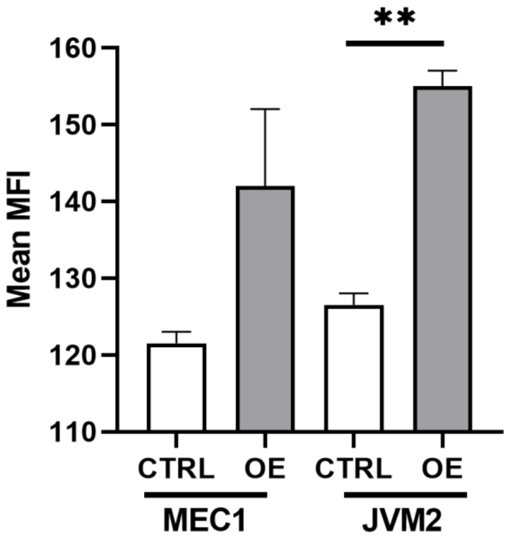

Figure S1. Intracellular calcium mobilization is higher in UGT2B17^{OE} leukemic cells. Leukemic cells (CTRL and overexpressing (OE) UGT2B17) were labeled with Fluo4-AM and basal calcium flux was measured by fluorescence activated cell sorting. Data are from 2 biological replicates conducted in duplicate analysis (mean +/- standard error of the mean are represented). MFI: mean fluorescence intensity.

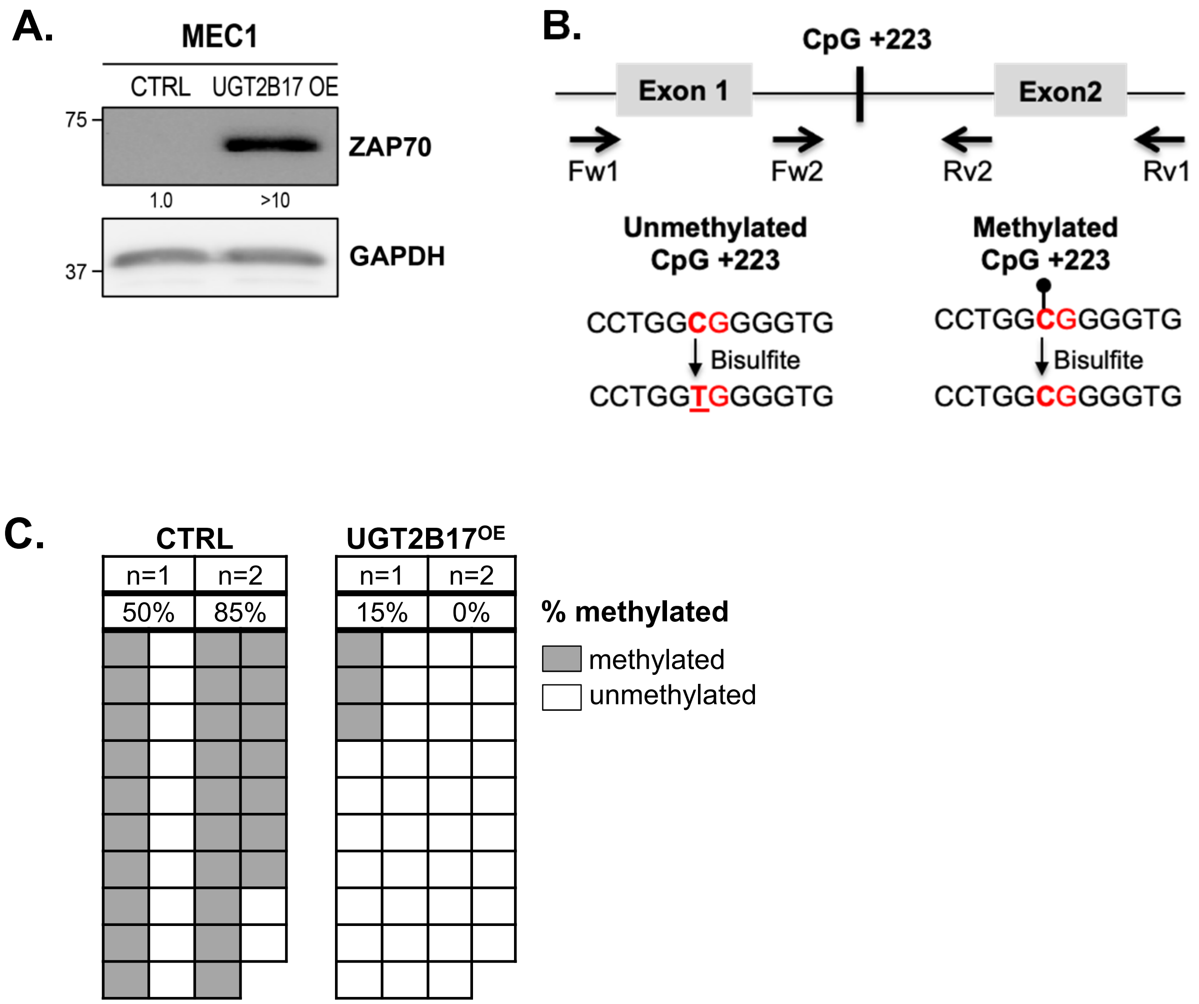

Figure S2. High UGT2B17 expression is associated with higher ZAP70 expression and a reduced methylation of the *ZAP70* 5' regulatory region. A. ZAP70 expression is strongly induced in MEC1 cells overexpressing UGT2B17 (UGT2B17^{OE}). B. The methylation status at the CpG dinucleotide located 223 nucleotides downstream of ZAP70 transcriptional start site was examined by a bisulfite treatment and nested PCR products amplified. C. Relative methylation status of the ZAP70 gene determined by sequencing cloned PCR products per cell model. Each square represent the methylation status of a cloned PCR product. Data is based on two independent replicate bisulfite assays.

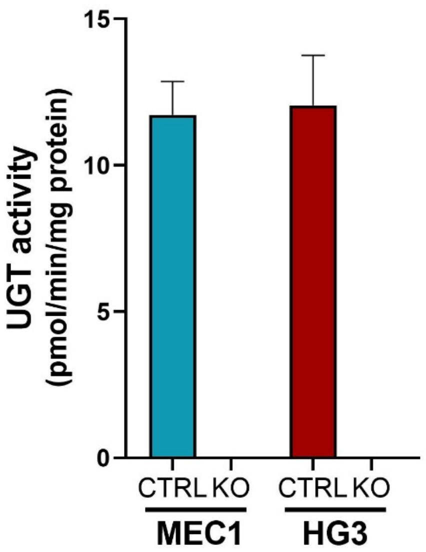

Figure S3. Validation of MEC1-UGT2B17^{KO} and HG3-UGT2B17^{KO} models by a specific enzymatic assay. A glucuronidation assay was conducted with homogenates of control (CTRL) and KO cell models using dihydrotestosterone as a UGT2B17-specific glucuronidation substrate. The UGT activity is abolished in the KO cell models

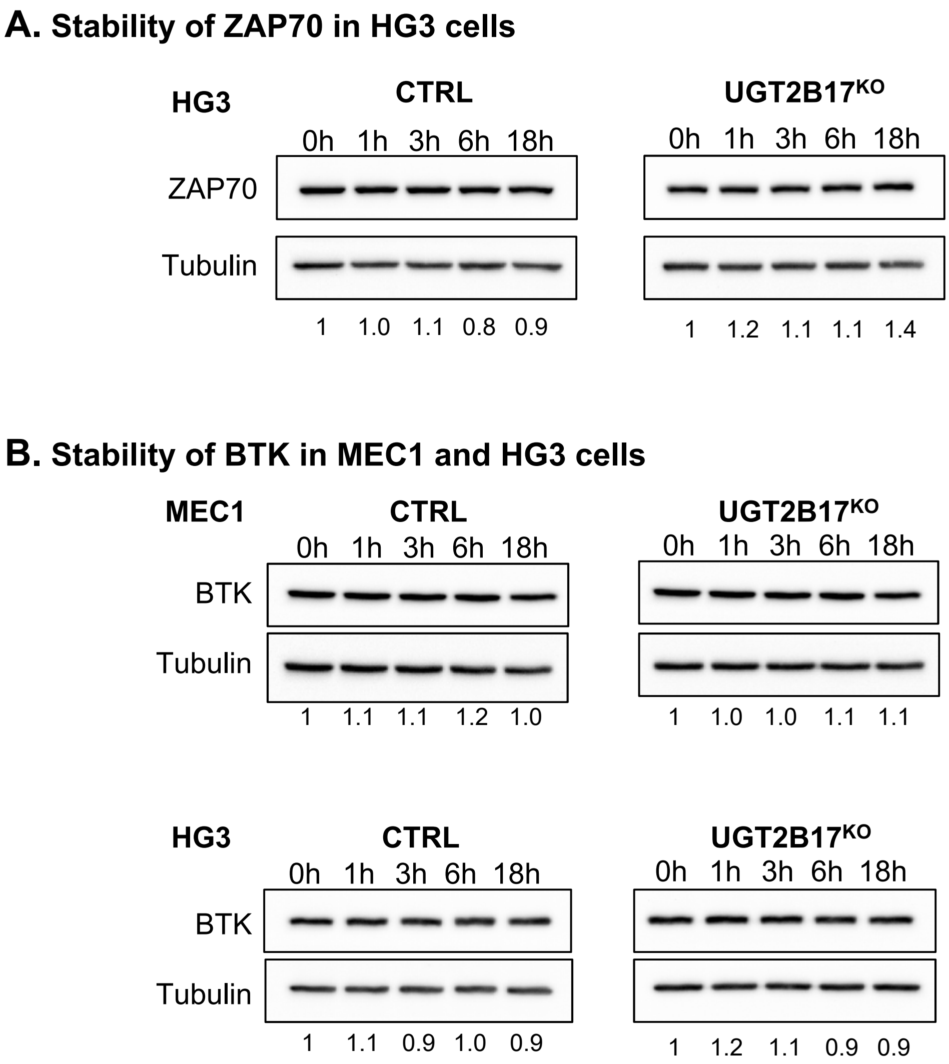

Figure S4. **The stability of BCR effectors is not compromised by the expression of UGT2B17.** The stability of (A) ZAP70 and (B) BTK was examined by translational inhibition with cycloheximide in control (CTRL) and UGT2B17 knockout (KO) cells. Protein expression levels were assessed by densitometry, and are given relative to the untreated samples below the immunoblots.
